# Organismal Function Enhancement through Biomaterial Intervention

**DOI:** 10.3390/nano14040377

**Published:** 2024-02-18

**Authors:** Fengchao Tian, Yuemin Zhou, Zaiqiang Ma, Ruikang Tang, Xiaoyu Wang

**Affiliations:** 1 Qiushi Academy for Advanced Studies, Zhejiang University, Hangzhou 310058, China; 22037047@zju.edu.cn (F.T.); zhou_yuemin@zju.edu.cn (Y.Z.); 2 Department of Chemistry, Zhejiang University, Hangzhou 310058, China; mzq2019@zju.edu.cn

**Keywords:** function enhancement, living organisms, biointerface engineering, artificial organelles, 3D multicellular immune niches

## Abstract

Living organisms in nature, such as magnetotactic bacteria and eggs, generate various organic–inorganic hybrid materials, providing unique functionalities. Inspired by such natural hybrid materials, researchers can reasonably integrate biomaterials with living organisms either internally or externally to enhance their inherent capabilities and generate new functionalities. Currently, the approaches to enhancing organismal function through biomaterial intervention have undergone rapid development, progressing from the cellular level to the subcellular or multicellular level. In this review, we will concentrate on three key strategies related to biomaterial-guided bioenhancement, including biointerface engineering, artificial organelles, and 3D multicellular immune niches. For biointerface engineering, excess of amino acid residues on the surfaces of cells or viruses enables the assembly of materials to form versatile artificial shells, facilitating vaccine engineering and biological camouflage. Artificial organelles refer to artificial subcellular reactors made of biomaterials that persist in the cytoplasm, which imparts cells with on-demand regulatory ability. Moreover, macroscale biomaterials with spatiotemporal regulation characters enable the local recruitment and aggregation of cells, denoting multicellular niche to enhance crosstalk between cells and antigens. Collectively, harnessing the programmable chemical and biological attributes of biomaterials for organismal function enhancement shows significant potential in forthcoming biomedical applications.

## 1. Introduction

Organisms, through the process of natural selection, have developed the capability to autonomously generate organic–inorganic hybrid materials, whether within their internal structures or externally. This process, also known as biomineralization, imparts additional protection or unique functionalities to the organisms [1]. For instance, shellfish produce hard shells that primarily comprise calcium carbonate to protect their soft bodies from predators, and single-celled diatoms create silica shells to shield themselves from ultraviolet radiation [2]. The occurrence of naturally formed hybrid materials inspires us to explore the enhancement of organisms through biomaterial intervention. This involves modifying organisms directly by designed biomaterials with diverse physicochemical properties to enhance their inherent functions and confer with new functions [3,4]. In addition to the formation of hybrid scaffolds within organisms, magnetotactic bacteria can synthesize a symbiotic organelle containing magnetic nanoparticles encapsulated within membrane compartment called a magnetosome to facilitate geomagnetic navigation [5]. Engineering organisms with biomaterials at the subcellular are also one of the most intriguing aspects for function enhancement [6]. For instance, Fe_3_O_4_ nanozymes with peroxidase-like activity can be integrated into the intracellular compartment of Paramecium (Para) to form multifunctional artificial organelles to endow Para with catalytic and magnetic responsive activities [7]. Accordingly, integration of smart-responsive material such as magnetic nanoparticles with unique and excellent properties may allow the organisms to respond to external inputs, such as chemical stimuli [8] (e.g., humidity and pH), mechanical stimuli [9] (e.g., stress, strain, and pressure), physical stimuli [10,11] (e.g., light, sound, and temperature), and electromagnetic stimuli [12] (e.g., electric field and magnetic field). However, due to the mechanisms of cell engulfment and processing of foreign substances, the construction of stable and long-lasting artificial organelles within cells remains challenging [13]. Aside from the regulation at the individual cell level, the development of hydrogel systems is driving the development of artificial microenvironments with tunable biochemical and biophysical properties for the construction and control of multicellular miniorgan-like structures [14]. Hydrogel materials can regulate cell recruitment, cell organization, and functionality of cell mass, providing engineering solutions for organism enhancement [15]. Implantation of a chitosan hydrogel in mice can create 3D multicellular immune niches by cell recruitment, where a variety of immune cells assemble to coordinate the local immune responses to further modulate the immune system [16]. Together, in contrast to genetic engineering that modifies organisms by changing their genome, biomaterial intervention represents a more convenient, safer, and reversible strategy that holds promise in applications to comprehensively enhance the functionality of organisms at various scales [17].

**Figure 1:**
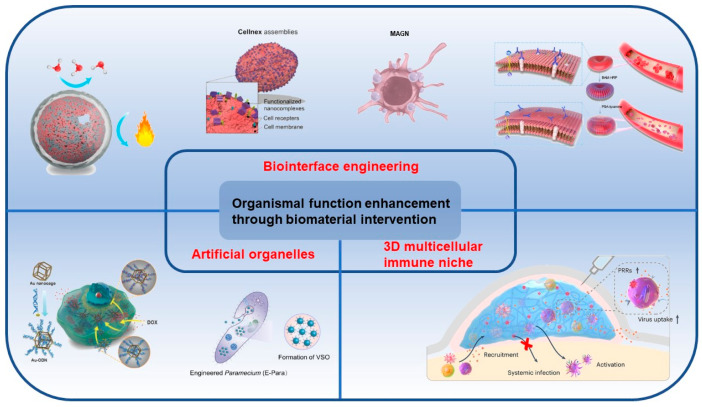
Three strategies to enhance organismal functions through biomaterial intervention, including biointerface engineering, artificial organelles, and 3D multicellular immune niches.

In this review, we focus on the organismal function enhancement through biomaterial intervention, and categorize the current strategies into biointerface engineering, artificial organelles, and 3D multicellular immune niches (Figure 1 and Table 1). In the second section on biointerface engineering, we introduce the engineering techniques for viruses and cells, emphasizing their significant implications in vaccine engineering, cell protection, cancer treatment, and blood transfusion. In the third section of artificial organelles, we discuss the fabrication of artificial organelle with natural enzymes and with nanozymes that are stabilized and protective by microcompartment-based strategy. Bio-enhancement through the introduction of artificial organelles into organisms covers a wide range of areas, including but not limited to cancer treatment, the creation of synthetic cells, responsive behavior to stimuli, and the clearance of waterborne viruses. The fourth section of this review focuses on 3D multicellular immune niches implanted within organisms, highlighting the strategy’s impact on the immune system and its crucial significance in cancer treatment. Finally, we also give a brief summary and outlook to provide a perspective on the current challenges and trends in the development of organismal function enhancement through biomaterial intervention.

## 2. Biointerface Engineering

Strategies for enhancing organism functionality are grounded in the chemobiological interactions between natural organisms and biomaterials, such as nanomaterials, mineral scaffold, and macroscale hydrogels. Among these strategies, one of the most prevalent methods involves the modification of biomaterial interfaces with shell scaffold [49,50]. As is well established, the interfaces of organisms, including individual cells and viruses, exhibit high modifiability, offering convenient binding sites for the assembly of biomaterials [51]. Within natural biointerfaces, amino acid residues, lipid membranes, or glycosylation sites of organisms, exhibiting charged, redox-active, and metal-coordinating reactivity, play a crucial role in facilitating the assembly of metal ions, minerals, and polymers to create material shells [52,53]. One of the major advantages of artificial shell-based intervention lies in the on-demand physicochemical properties of shell structure that can remodel the interaction of organisms with external environment, interfacial recognition, and signal exchange, facilitating functionalities such as cell protection [29], stimuli-responsive shells [54], and camouflage [33].

The surfaces of cells and viruses are rich in biomacromolecules that provide net negative charges. This has inspired biointerface engineering, connecting non-biological materials to the biointerface [3]. Examples of natural biomineralization include oviparous animals protecting embryos with eggshells and diatoms producing silica shells for UV protection [2]. Mimicking biomineralization to form robust shells on the surface of biological entities is one of the most attractive strategies in biointerface engineering [55,56]. Additionally, non-mineral shells play crucial roles. For instance, spores formed by *Bacillus subtilis* have tough multilayered shells that comprise peptidoglycans and proteins to withstand adverse environments like nutrient deprivation [57]. In summary, encapsulating individual viruses or cells through biointerface engineering can effectively enhance the biological performance of living organisms. In this section, we will review the development of biointerface engineering based on the differences in the structures of viruses and cells.

Viruses comprise proteins containing numerous aspartic acid and glutamic acid residues [58]. Metal cations can easily bind to viruses with negative charges through electrostatic interactions, promoting the formation of inorganic mineral layers. Researches have shown that the supersaturated solution of metal ions can lead to spontaneous biomineralization of viruses. Silicified viruses are found in high-salt lakes and hot springs exhibiting a mineralized shell [59]. Analogously, viruses can undergo in situ mineralization to produce calcium phosphate (CaP) shells, making them more robust and capable of infecting human host cells through non-receptor-dependent pathways [60]. Since 1999, many studies have also focused on using plant virus, such as tobacco mosaic virus (TMV), as a template to generate nanostructures like iron oxide and silica nanotubes [61,62].

One of the purposes of virus encapsulation is to enhance the thermal stability of the virus, reducing the dependence of virus vaccines on the cold chain. The World Health Organization (WHO) estimates that nearly 50% of freeze-dried vaccines are discarded each year [63]. The lack of cold chain infrastructure is a significant factor contributing to the spread of infectious diseases in impoverished countries. Our group’s work indicates that inducing the formation of CaP mineral shells on the surface of Japanese encephalitis virus (JEV) and human enterovirus 71 (EV71) can allow storage for over a week at 37 °C [18,19]. Another approach involves forming a hydrated silica layer on the virus surface, creating a structured hydrated layer covering the virus’s exterior, reducing heat conduction to the virus’s interior [64,65]. In addition to in situ mineralization, utilizing electrostatic interactions to aggregate positively charged aluminum oxide nanoclusters around virus particles, forming an aluminum oxide gel-like nanocoating, can also significantly enhance their thermal stability [20].

Another role of virus encapsulation is to create a disguised outer shell to overcome pre-existing immunity. Recombinant adenovirus serotype 5 (rAd5-Env) vectors expressing simian immunodeficiency virus (SIV) envelope protein undergo in situ biomineralization in a culture medium rich in Ca^2+^ [66]. The resulting vaccine-material hybrid possesses a biodegradable core-shell structure, which can mask the vaccine surface while preserving its original activity. This shielding vaccine can evade pre-existing anti-Ad5 immunity, enhancing Env-specific T cell responses. The disguise strategy can also be employed to eliminate antibody-dependent enhancement (ADE) effects in dengue virus infection, significantly improving the safety of dengue fever vaccines [21]. Furthermore, viruses encapsulated with CaP mineral layers exhibit higher and more prolonged transfection activity in vivo [67]. The CaP mineral layer also alters the physical and chemical properties of the original vaccine, leading to increased adhesion to nasal mucosa, facilitating the development of needle-free intranasal vaccine administration [68].

**Figure 2:**
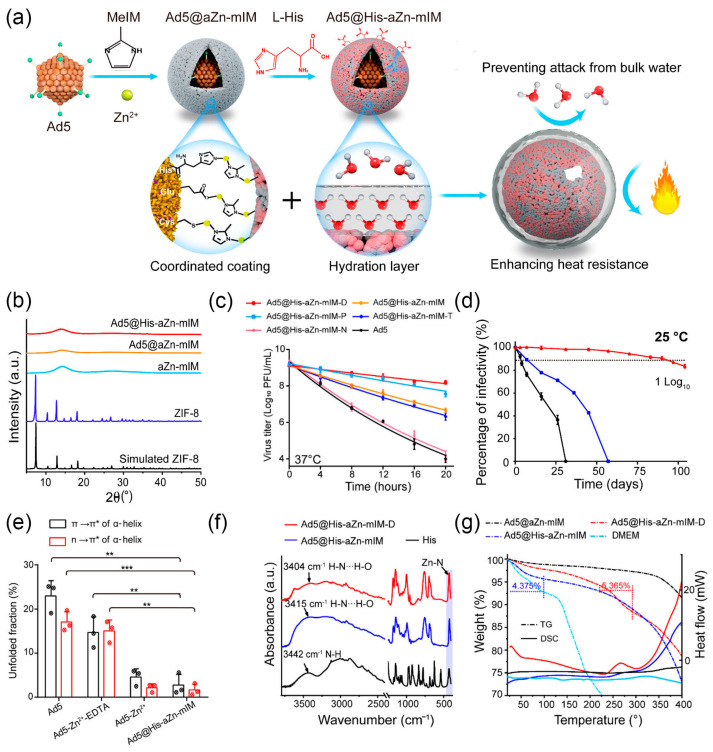
(**a**) Schematic diagram of Ad5@His-aZn-mIM. (**b**) Characterization using pXRD patterns. (**c**) Thermal inactivation kinetics of Ad5@His-aZn-mIM in different biological solutions after being stored at 37 °C (*n* = 5). (**d**) The remaining percentages of infectivity at 25 °C over time. (**e**) Comparison of the unfolded degrees of the π → π* and *n* → π* transitions of the α-helix following treatment at 25 °C for 6 h. (n = 3, * *p* < 0.05, ** *p* < 0.01, *** *p* < 0.001). (**f**) FT-IR spectra. (**g**) TG (dashed line) and DSC (solid line) curves in N_2_. Reprinted with permission from Ref. [22]. Copyright (2022) American Chemical Society.

Compared to inorganic mineral layers, metal-organic frameworks (MOFs) come with the added benefits of highly adjustable design and ease of surface modification [69]. Among all MOFs, ZIF-8 has been selected as the encapsulation material in numerous studies due to its ability to be synthesized and remain stable under aqueous conditions [70]. In 2016, the Qu group pioneered an MOF-based vaccine by encapsulating ovalbumin (OVA) within ZIF-8 nanoparticles and further attaching cytosine-phosphate-guanine oligodeoxynucleotide (CpG ODN) on the particle surface [71]. Additionally, ZIF-8 was employed to construct a protective shell on the surface of TMV. By adjusting the ligand-to-metal ratio during shell growth, shells of different thicknesses were obtained [72]. In subsequent in vivo experiments, ZIF-8-encapsulated TMV was proven to maintain integrity, biocompatibility, and immunogenicity, providing a method for simultaneously protecting and delivering protein drugs [73]. Zhang et al. use ZIF-8 to construct a nanocoating for Ad5 to enhance vaccine thermal stability and eliminate the need for a cold chain (Figure 2a) [22]. However, the challenge lies in the alkaline conditions during ZIF-8 synthesis, crucial for deprotonating 2-methylimidazole (2-mIM) into a Lewis base, but this may potentially deactivate the Ad5 vaccine [74]. To address this issue, the authors built an amorphous Zn-mIM coordination polymer layer (Ad5@aZn-mIM) on the Ad5 surface at lower 2-mIM to zinc nitrate ratio (Figure 2b). Importantly, by partially replacing imidazole ester linkers with histidine (His), the resulting Ad5@His-aZn-mIM demonstrated significantly enhanced stability in biological buffers such as phosphate-buffered saline (PBS) and culture media (Figure 2c). Results showed that Ad5@His-aZn-mIM effectively extended the efficacy of Ad5 vaccine at room temperature (with less than 1 log10 PFU titration loss) for up to 90 days without the need for a cold chain (Figure 2d). Mechanistic studies indicated that the nanocoating on the Ad5 surface stabilized the rigid conformation of the virus protein’s α-helix (Figure 2e). The polar groups in His strengthened hydrogen bond formation with solvent water molecules, creating a hydrated shell on the Ad5@His-aZn-mIM surface, further enhancing its conformational stability at high temperatures (Figure 2f,g). Recently, Wang et al. successfully synthesized crystalline ZIF-8 on foot-and-mouth disease virus by pre-lowering the pH of the 2-mIM solution to 9.0 [75]. The encapsulated viral vaccine induced approximately ten times higher antibody titers and significantly increased cellular immune responses in mice.

Aside from viruses, the encapsulation of cells, which is enveloped by a fluidic lipid bilayer membrane containing 40% proteins, requires more sophisticated design, as cells are highly sensitive to chemical treatments and susceptible to high levels of osmotic pressure and mechanical stress [76]. Yeast cells, algae, and some prokaryotic microorganisms have cell wall structures that increase their tolerance to mechanical stress. However, in the case of yeast cells, their cell wall primarily comprises mannose and N-acetylglucosamine [77]. This structure hardly induces templated crystallization of minerals due to its relatively low charge density. Therefore, to achieve optimal shielding and protection for cells, it is necessary to employ a polymer-assisted approach to induce the formation of mineral shell. Only a few examples have directly deposited inorganic shells of calcium carbonate [78] and manganese dioxide [79] on the surface of yeast cells. Additionally, the encapsulation of living cells requires a thorough assessment of the impact of the shell on cell proliferation and the exchange of substances between the cell and the external environment. The former requires the shell material to be either thin and flexible or degradable to release viable cells in specific environments. The latter imposes requirements on the permeability of the shell.

In 2007, Lvov pioneered the encapsulation of mesenchymal stem cells (MSCs) using layer-by-layer self-assembly (LbL) technology [80]. Through LbL, the negatively charged cell membrane can be wrapped in a cationic polymer shell, and polymers with opposite charges can be sequentially adsorbed to achieve the desired thickness and surface composition [81]. The shell’s permeability can be altered based on composition and layer numbers. Owing to the controllability of thickness, permeability, and stiffness, LbL films have played a crucial role in the development of cell encapsulation technology. The encapsulation of neural stem cells (NSCs) using LbL shows no effect on cell viability, proliferation, or differentiation [82]. To endow the regulatory function of LBL shell, insulin-like growth factor-1 (IGF-1) can be loaded into the coating, enabling time- and pH-dependent release. LbL encapsulation of MSC can be used for intravenous administration to extend their circulation lifespan and show increased accumulation [83]. LbL has also been used to design enzyme-responsive polymer nanoshells. By connecting different polymer layers with a peptide chain sensitive to matrix metalloproteinase-7 (MMP-7), an enzyme overexpressed in most tumors, encapsulated MSCs can achieve selectively accumulation in tumor areas [23].

LbL layer also facilitates further deposition of mineral layers to form a hybrid shell that helps cells avoid harmful substances from the external environment, such as trypsin and ultraviolet radiation [84]. In 2008, our research group first described the in situ synthesis of egg-shell-like structures onto the surface of yeast cells, and explored the possibility of using a biomimetic mineral shell to protect cells from harsh conditions [24]. Using a similar approach, yeast cells [85] and several mammalian cells [25] were also successfully encapsulated in silica coatings. The physical non-permeability of the silica coating rendered human cervical cancer (HeLa) cells unprecedented resistance to lethal trypsin and polyvinylamine hydrochloride [25]. However, due to the non-degradable nature of silica, the cells inside eventually died. One of the solutions to maintain cell survival and proliferation is forming a semi-encapsulated silica shell on adherent cells [86]. Youn et al. designed a TiO_2_ shell for encapsulating Jurkat T cells, which did not affect cell division and biological activities such as cytokine secretion [26]. In addition to its role in cell protection, LnPO_4_ shells on zebrafish embryos can protect the embryos from UV radiation [87]. During the silicification of green algae, the cell-material aggregate forms and creates an anaerobic environment, which activates hydrogenase and significantly increases hydrogen production [27]. Following a similar research approach, constructing a sandwich-like layer containing laccase outside a single Chlamydomonas cell triggers hydrogen production by creating an anaerobic environment inside the cell [88].

**Figure 3:**
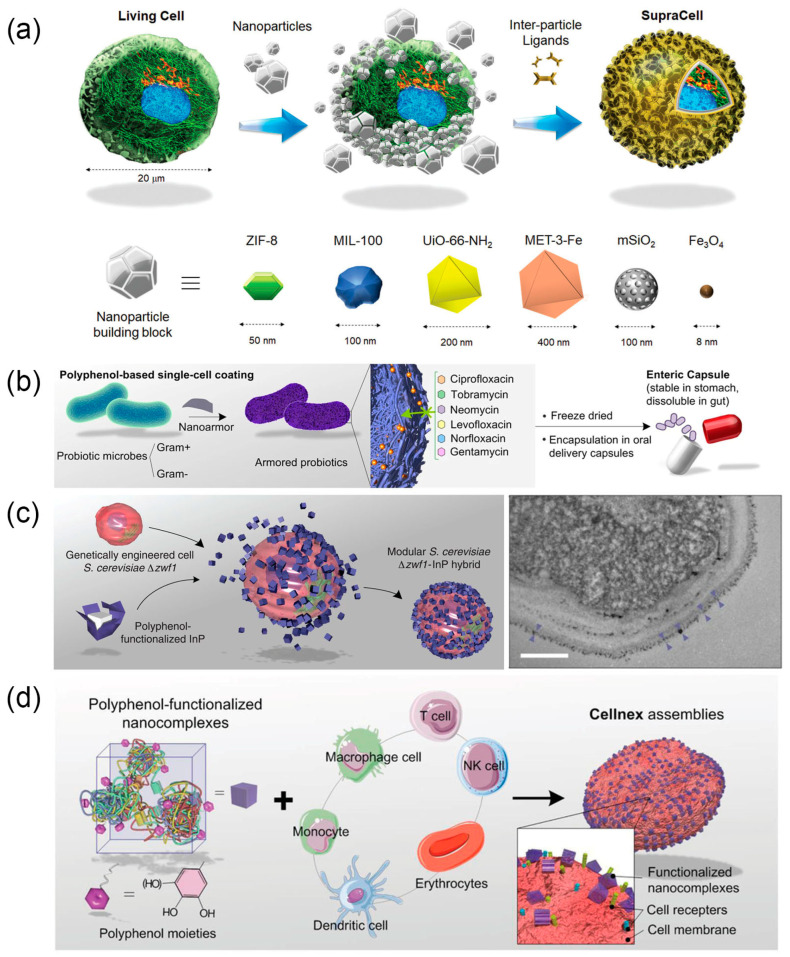
(**a**) Schematic representation of TA-assisted formation of NP, including MOF NP, shells on individual living cells, leading to the generation of SupraCells. Reprinted with permission from Ref. [91]. Copyright (2019) Wiley-VCH (Weinheim, Germany). (**b**) The nanoarmor enables a rapid and highly biocompatible single-cell encapsulation that protects from a wide range of antibiotics. Reprinted with permission from Ref. [29]. Copyright (2022) Springer Nature. (**c**) Schematic of assembly (**left**) and TEM image (**right**) of *S. cerevisiae*–InP hybrids. The purple triangles highlight the positions of individual InP nanoparticles. Scale bar: 500 nm. Reprinted with permission from Ref. [92]. Copyright (2018) American Association for the Advancement of Science. (**d**) Modular assembly of “Cellnex” through the assembly of polyphenol-functionalized biologically active nanocomplexes on cells. Reprinted with permission from Ref. [93]. Copyright (2020) Wiley-VCH.

MOF-based shell with high porosity allow for the transport of nutrients, maintaining cell activity [89]. MOF shells can be easily removed with EDTA or acidic pH to fully restore the cell’s original function [90]. Loading β-galactosidase into the pores of ZIF-8 imparts yeast cells with the ability to catalyze lactose into glucose, enabling survival in a simulated extreme nutrient-depleted environment [28]. Zhu et al. introduced the concept of “SupraCells”, encapsulated and protected living mammalian cells within an exoskeleton based on functional modular nanoparticles, which provide an ability to endow the encapsulated cell with useful, tunable physicochemical properties (e.g., optical, magnetic, and sensing properties) depending on the NPs or NP combinations (Figure 3a) [91]. Colloidal solutions of building blocks such as ZIF-8 were mixed with live cells and quickly adhered to their surfaces. Subsequently, under the action of inter-particle ligands, such as tannic acid for MOF systems based on metal–phenolic interactions, the nanoparticles quickly combined to form a coherent, stable shell. The entire process was completed in a very short time, avoiding the typical internalization pathway of nanoparticles.

Metal-polyphenol networks (MPNs) can be coated onto various interfaces in a one-step process due to the general surface binding affinity of TA [94]. Fe^3+^ and tannic acid (TA) can form a nano-thin film shell on the surfaces of yeast cells [95] and mammalian cells [96]. This thin film exhibits pH-dependent degradation, allowing for controlled cell encapsulation and release [94]. Additionally, they can form “nano-armored” structures on the surface of intestinal probiotics, demonstrating strong colonization ability in the gastrointestinal tract of mice and significantly reducing antibiotic-associated diarrhea (Figure 3b) [29]. Apart from the thin film shell, Guo et al. have established a method to modularly assemble polyphenol-functionalized components onto cells to construct complex micro/macro hybrid materials [97,98]. They have developed a *S. cerevisiae*–indium phosphide (InP) hybrid system (Figure 3c) [92]. Polyphenol-functionalized photosensitive InP particles are assembled onto genetically engineered yeast cells, imparting the yeast cells with the capability to capture photoelectrons for reductive biosynthetic reactions. Similarly, using this approach, polyphenol-functionalized biomolecules such as functional proteins, DNA, and virus vectors can be assembled on the surfaces of various mammalian cells (Figure 3d) [93]. This cell engineering platform, named “Cellnex”, is highly customizable, allowing for rapid preparation in less than 10 min, and holds promise for applications in cell-based therapies and biohybrid cell engineering.

Inspired by mussels, live yeast cells [99] and dendritic cells (DCs) [100] have been successfully encapsulated in functionable polydopamine (PDA) shells. The behavior of the cells is controlled by the thickness of the shell while maintaining cell viability. Fe^3+^ and benzene-1,3,5-tricarboxylic acid (BTC) can form degradable extracellular shells onto cells. Importantly, by simply adding enzymes to the BTC precursor solution, various enzymes can be embedded in the nano shell, achieving multi-enzyme cascade reactions [101]. Subsequent research indicated that incubating Fe^3+^-BTC in PBS for 24 h led to its transformation into a more robust Fe^3+^-P shell [54]. Although cell viability decreased, this transformation released bioactive molecules from the shell, demonstrating enhanced cell functionality. Hydrogen-bonded organic frameworks (HOFs) have also been used to encapsulate neural stem cells (NSCs) followed by loading porous carbon nanospheres (PCNs) in the network [102]. The HOF shell prevents the loss of NSC stemness before transplantation and reduces cell membrane damage during the transplantation process. PCNs have excellent light absorption capability and high photothermal conversion efficiency in the infrared window [103]. After transplantation into the mouse hippocampus, irradiation with 1064 nm infrared light induces thermal response, leading to hydrogen bond dissociation and releasing NSCs, achieving therapeutic functions.

**Figure 4:**
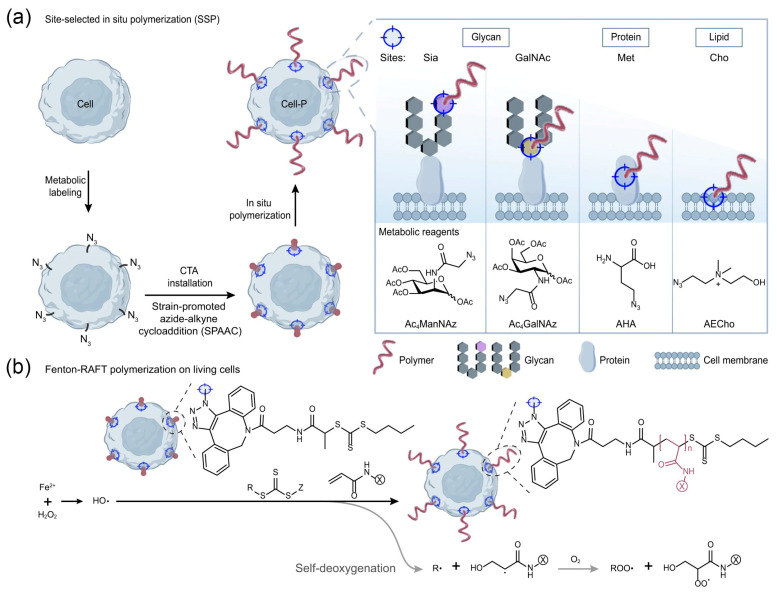
(**a**) Confinement of the chain transfer agent (CTA) site by metabolic labeling allows polymerization to occur at selected glycan (sialic acid, Sia; GalNAc), protein (methionine, Met), and lipid (choline, Cho) sites on the cell surface. (**b**) Proposed mechanism of the living cell surface-initiated, self-deoxygenating Fenton-RAFT polymerization reaction. Reprinted with permission from Ref. [30]. Copyright (2023) Springer Nature.

Polymer grafting is one of the strategies for living cell biointerface engineering. Controlled free radical polymerization to introduce high-density functional polymers on cell surfaces has gained widespread attention [104,105]. In this technique reported by Niu et al., 2-(butylthiocarbonothioyl) propionic acid (BTPA), as the chain-transfer agent (CTA), is introduced onto cell surfaces using either covalent (yeast) or non-covalent insertion strategies (mammalian) [106]. Subsequently, polymerization is initiated through photoinduced electron transfer-reversible addition-fragmentation chain-transfer polymerization (PET-RAFT), grafting various functional polymers onto cell surfaces. Inspired by this research, Zhong et al. recently reported an in situ controlled free radical polymerization platform for precisely controlling the grafting sites of polymers on live cell surfaces [30]. Firstly, biologically orthogonal groups are introduced at different sites on the cell membrane (glycans, proteins, lipids) through metabolic labeling for CTA installation (Figure 4a). Fenton-RAFT polymerization is then conducted to in situ construct polymers at selected sites (Figure 4a,b). The cell skeleton and various physiological functions of Jurkat T cells are well-preserved after polymerization. Polymers grown at different sites can confer T cells with resistance to lectin-induced cell apoptosis.

In addition to the typical use of cell shells for cell protection or cargo loading, there are some creative applications. A pathological mineralization that kills cancer cells called drug-free cancer cell targeted calcification (CCTC) [31] is proposed by the Tang group. Folate receptors (FRs), upregulated in many human tumors but expressed at lower levels in normal cells [107], can specifically bind to folate (FA) molecules, leading to accumulation of carboxylate residues of FA on the interface of tumor cells. Carboxylate residues of FA selectively bind Ca^2+^ in biological fluids, promoting calcium mineral nucleation. Thus, it can be reasonably expected that the abundant FR on cancer cells can be utilized for cancer cell-targeted calcification. Both in vitro and in vivo experiments validated that Ca^2+^ and folate could selectively calcify tumor cells, inhibiting tumor growth. To address the issue of high concentrations of Ca^2+^ required for this method, which may induce hypercalcemia, Tang et al. designed a new polysaccharide-based conjugate combining FA and polysialic acid [108]. FA targets tumor cells, while polysialic acid provides multiple carboxylic acid groups to sequester calcium from the blood, selectively inducing cancer cell calcification.

**Figure 5:**
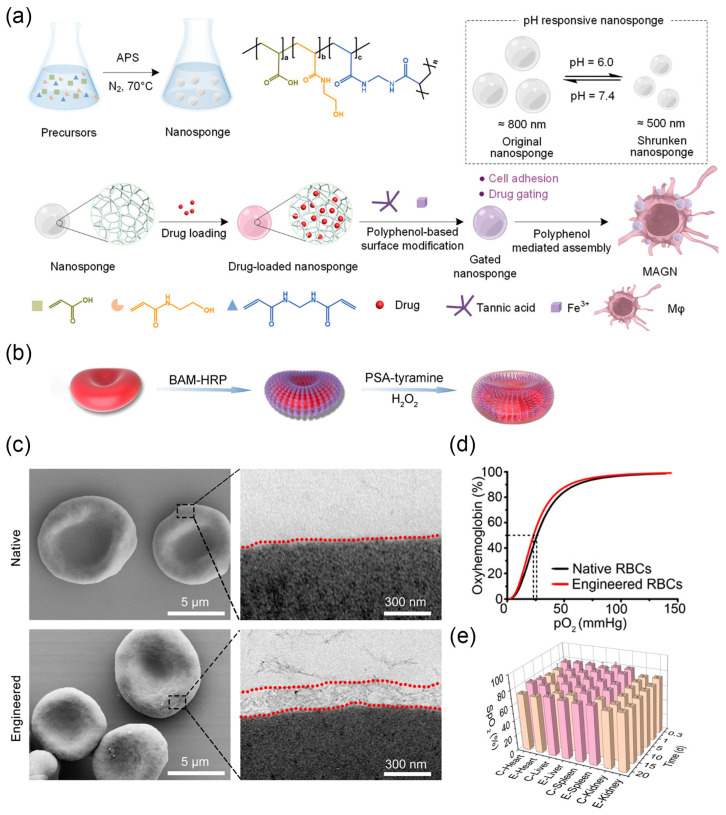
(**a**) Stimuli-responsive nanosponges capped with metal-phenolic supramolecular gatekeepers anchored on tumor-targeting Mφ (MAGN platform) against metastatic tumors. Reprinted with permission from Ref. [32]. Copyright (2023) Wiley-VCH. (**b**) Schematic illustration of the procedure for RBC surface engineering. (**c**) SEM images (**left**) and TEM images (**right**) of human native RBCs and surface-engineered human RBCs. (**d**) Structural and functional analysis of the native RBCs (black) and engineered RBCs (red). (**e**) Quantitative intensity of oxyhemoglobin in the liver, spleen, and kidney after injection. Reprinted with permission from Ref. [33]. Copyright (2020) American Association for the Advancement of Science.

Cell-based drug delivery systems leverage the tropism of live cells to efficiently infiltrate inflamed tissues with remarkable specificity, achieving effective targeted drug delivery [109]. Liao et al. reported a macrophage-driven automatic homing of gated nanosponges, referred to as MAGN (Figure 5a) [32]. A high content of therapeutic drugs is securely encapsulated within nanosponges capped with metal-phenolic supramolecular gatekeepers. The nanosponges are then integrated on the surface of tumor-homing macrophages. After the engineered macrophages homed to the tumor region [110], the significant contraction of Fe^3+^-TA gatekeepers and nanosponges in the acidic tumor microenvironment allowed for controlled drug release at the targeted site. Due to the highly porous structure of the designed nanosponges, the MAGN platform may serve as a versatile platform to achieve high loading and enhanced delivery of various therapeutic drugs (e.g., DNA/RNA, monoclonal antibodies, and peptides) to the desired pathological site. In addition to tumor-homing macrophages, red blood cells administered intravenously can preferentially co-localize in the lungs, making them suitable for designing cell-based drug delivery systems [111]. Chemotactic factors anchored on the surface of injected red blood cells induce immune cell infiltration into the lungs, inhibiting the progression of lung metastatic tumors and achieving systemic immunity [112]. Adeno-associated virus (AAV) can also be anchored on the surface of red blood cells for delivery to the lungs, achieving high-intensity targeted gene expression in the lungs and successful transgene expression upon re-administration [113].

By masking the Rhesus D (RhD) antigenic sites on red blood cells (RBCs), a process known as “invisibility”, universal RBCs can be prepared for emergency blood transfusions. RhD is one of the most important immunogenic antigens on RBCs. However, as a rare blood type, RhD-negative blood for transfusions is severely scarce and in extreme shortage, especially in emergency situations [114]. Using chemically polymerized disguises to encapsulate RBCs is a viable strategy to avoid immune reactions [115]. Although some biomimetic molecules (such as PDA [116]) can achieve antigenic site masking on RBCs, this treatment often leads to a significant reduction in membrane fluidity, making the modified RBCs prone to cell fragility and destruction. To address this issue, Zhao et al. combined the biocompatible anchoring molecule BAM and horseradish peroxidase (HRP) onto the surface of RBCs [33]. Subsequently, HRP catalyzed the polymerization of polysialic acid (PSA) and tyramine, forming a gel-like network layer about 250 nm thick on the surface of RBCs (Figure 5b,c). As a result, the antigenic determinants of RBCs were masked, preventing recognition by the immune system. Engineered RBCs maintained membrane fluidity and cell deformability. The oxygen dissociation curve (ODC) of hemoglobin in natural RBCs and engineered RBCs showed almost no difference, indicating similar oxygenation performance (Figure 5d). After in vivo transfusion, natural RBCs and engineered RBCs exhibited similar oxygenated hemoglobin levels in the same organ at different time points (Figure 5e). Subsequent in vivo experiments in rabbit models demonstrated that engineered RBCs induce no immune rejection.

## 3. Artificial Organelle

In addition to the extracellular shell, material engineering at the subcellular level, specifically the creation of artificial organelles, plays a distinct role in orchestrating the overall activities of organisms. In this section, we initially introduced the significance of artificial organelles, their design methods, and distinctions from drug delivery systems. Subsequently, we progressively discussed the research progress of artificial organelles, divided into compartmentalized natural enzymes, individual nanozymes, and compartmentalized nanozymes.

Resembling natural cellular organelles, artificial organelles refer to nanoreactors that are fabricated through synthesis or chemical engineering. They are designed to simulate, replace, or enhance specific functions within cells to adjust cell deficiency, enhance function, or endow cells with new capabilities [117]. Currently, artificial organelles can be introduced into cancer cells to locally convert low-toxicity prodrugs into therapeutic drugs [118,119], or pre-introducing artificial organelles into normal cells to eliminate unexpected drug accumulations [38]. Furthermore, research on artificial organelles lays the groundwork for the bottom-up construction of synthetic cells [120].

The design of artificial organelles should be comprehensively addressed from three aspects: functional design, material selection, and structural design. Firstly, the function of artificial organelles must be defined, which may include catalyzing reactions [121,122], molecular storage and release [123], as well as thermal and magnetic effects [12,124]. Next, appropriate materials must be selected, with a focus on good compatibility and stability in subcellular environments, enabling the controlled execution of the desired functions. Lastly, the structural design of artificial organelles needs to account for factors such as shape, size, and intracellular localization, as these aspects affect the organelles’ ability to enter cells and escape from lysosomes [125].

In eukaryotic cells, one of the most classical features is the compartmentalized structures formed by the cell membrane (e.g., lysosomes and mitochondria), creating separated environments between the internal and external regions. Inspired by these constructions, the most intuitive approach to construct an artificial organelle involves loading natural enzymes into artificially engineered compartmental structures. In this concept, the challenge shifts to the construction of microcompartment structures. It is essential to emphasize that these microcompartment structures differ significantly from nanocarriers used in drug delivery. Nanocarriers for drug delivery aim to isolate the external environment from contact with the drug and undergo structural breakdown in lysosomes to release the carried drug. In contrast, microcompartment structures for artificial organelles must strike a delicate balance between structural stability and permeability to ensure substrate entry and product release. Additionally, their structure must remain intact during the process of escaping from lysosomes to the cytoplasm [126,127,128].

The fragile natural enzymes need to be gently loaded into microcompartment structures to construct artificial organelles. Therefore, materials for microcompartments typically include polymers [129], liposomes [130], porous silica nanoparticles [131], and metal-organic framework [132]. The advantages and disadvantages of these materials have been extensively studied in the past [133,134]. Controllable morphology porous silica nanoparticles with high surface area and porosity can provide good permeability for loading enzymes [135]. Chang et al. reported a novel one-pot synthesis of HRP encapsulated hollow silica nanospheres (HRP@HSN) based on templating reverse microemulsions [118]. Small HRP@HSN (diameter ~50 nm) exhibited satisfactory catalytic activity and served as an intracellular catalyst for oxidizing the prodrug indole-3-acetic acid, generating toxic free radicals to kill cancer cells [34]. Liposomes and polymers can be used to easily construct stable microcompartment structures, crucial for building subcompartmentalized artificial organelles. Thingholm et al. used silica particles as a core, deposited a layer of liposome subunit loaded with glucose oxidase (GOx) on its surface, and further modified the surface with a polymer [35]. Macrophages internalizing this artificial organelle exhibited reduced vitality when exposed to glucose due to hydrogen peroxide production. Godoy-Gallardo deposited layers of liposome subunits loaded with trypsin (TRP) and horseradish peroxidase (HRP) on the surface of silica particles, separated by polymer layers [36]. Macrophages internalizing this artificial organelle were capable of simultaneously undergoing two enzyme-catalyzed reactions.

From a different perspective, nanozymes, as substitutes for natural enzymes, can also be employed to build stable artificial organelles. Recently, Zhang et al. comprehensively reviewed the latest progress in artificial organelles based on nanozymes, discussing their potential applications and related challenges in biomedicine [6]. Nanozymes are nanomaterials that catalyze the conversion of enzyme substrates to products, following enzyme kinetics, in physiological environments. In 2007, Gao et al. first reported that Fe_3_O_4_ nanoparticles possess intrinsic enzyme-mimetic activity similar to natural peroxidases, sparking extensive and in-depth research on nanozymes [136]. In contrast to natural enzymes with high catalytic selectivity, one characteristic of nanozymes is their multiple enzyme activities, facilitating the construction of cascade reactions. Importantly, due to the properties of nanomaterials, most nanozymes can withstand extreme environmental conditions (high temperature, extreme pH, and organic solvents), ensuring minimal loss of activity during the construction and application of artificial organelles.

**Figure 6:**
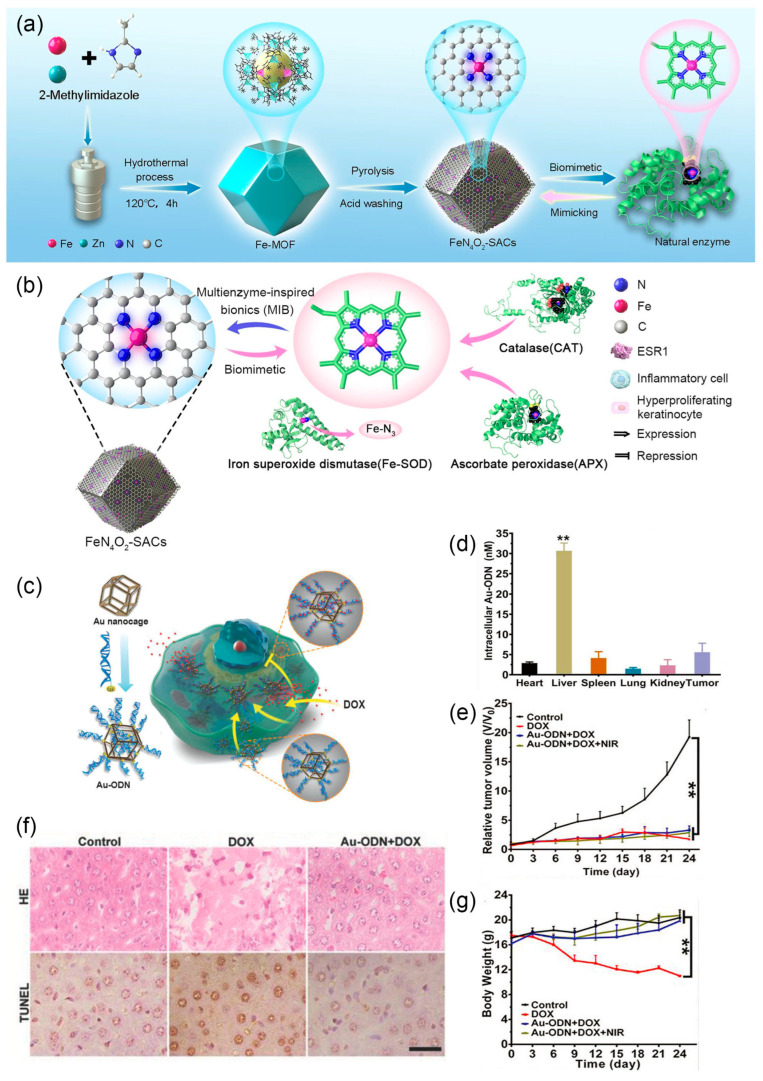
(**a**) Synthetic process of biomimetic FeN_4_O_2_-SACs. (**b**) Schematic illustration of FeN_4_O_2_-SACs for psoriasis catalytic therapy. Reprinted with permission from Ref. [137]. Copyright (2023) Springer Nature. (**c**) Schematic of the construction of Au-ODN and its working principle as a nanomaterial-based organelle within the cell. (**d**) Average Au-ODN concentration within each organ’s cells after injection (** *p* < 0.01). (**e**) Normalized tumor growth curves (** *p* < 0.01). (**f**) H&E staining and TUNEL analysis of liver slices, the black bar represents 25 µm. (**g**) Body weights of mice after the different treatments (** *p* < 0.01). Reprinted with permission from Ref. [38]. Copyright (2018) Wiley-VCH.

The outstanding stability allows nanozymes to enter cells and function as an artificial organelle without protection of microcompartment. Liu et al. prepared ultra-small RuO_2_ nanoparticles (average size 2 nm) as antioxidants for acute kidney injury [37]. These nanoparticles can mimic the activity of catalase (CAT), peroxidase (POD), superoxide dismutase (SOD), and glutathione peroxidase (GPx), eliminating excess reactive oxygen species (ROS) and significantly reducing ROS-induced cell apoptosis. After intravenous injection into mice, ultra-small RuO_2_ nanoparticles significantly inhibited the development of acute kidney injury in mice. Similarly, to treat exacerbated psoriasis due to ROS overexpression, Lu et al. demonstrated the use of biomimetic iron single-atom catalysts (FeN_4_O_2_-SACs), which can mimic the activities of CAT, SOD, and ascorbate peroxidase (APX), effectively improving psoriasis-like symptoms in mice and preventing recurrence (Figure 6a,b) [137]. Researchers drew inspiration from the structure of active sites in natural antioxidant enzymes, where active iron is connected to four nitrogen atoms. They ingeniously prepared iron-doped zinc metal-organic framework (ZIF-8) precursors, which, after high-temperature pyrolysis and acid washing, resulted in FeN_4_O_2_-SACs (Figure 6a) [138]. On the other hand, the strategy of increasing ROS can also be used to selectively kill tumor cells. Nanozymes based on Ag, Pt, Pd, Fe, and Mn enhance the production of hydroxyl radicals (·OH) through combined use. Combined with photodynamic therapy (PDT), photothermal therapy (PTT), and starvation therapy, they achieve synergistic anti-tumor treatment, providing a new strategy for cancer therapy [139,140,141,142]. Currently, chemotherapy remains one of the main approaches for treating cancer. However, despite the widespread use of targeted drug delivery, over half of the chemotherapy dose still concentrates in normal tissues, especially the liver [143]. As mentioned earlier, the use of prodrugs is one strategy to reduce the toxicity of chemotherapy drugs. Zhao et al. approached the elimination of unexpected cytotoxicity in normal cells from another aspect (Figure 6c) [38]. Previous studies suggested that double-stranded oligonucleotides (ODNs) rich in GC can bind to the chemotherapy drug doxorubicin (DOX) [144]. The authors used gold nanocages as a matrix material to immobilize ODN to protect it from degradation by nucleases. The resulting Au-ODN selectively accumulated in liver cells when circulating in the body (Figure 6d). The subsequent intravenous injection of DOX indicated that, while maintaining its anticancer effects (Figure 6e), this artificial organelle could effectively protect the liver and reduce side effects (Figure 6f,g).

Although nanozymes are stable enough in physiological environments, loading them into microcompartment structures is still necessary, which can create a local microenvironment or chemical gradient, aiding in the positional assembly and enrichment of catalytic substances to ensure processes proceed with higher efficiency. Xu et al. designed an “artificial mitochondria” structure mimicking oxidative phosphorylation [145]. The authors immobilized gold nanoparticles (AuNPs) with dual enzyme activities (GOx and POD) in hollow silica microspheres (HSMs) and further deposited liposome subunits loaded with ATP synthase on the microsphere surface. In the presence of oxygen and catalysis by AuNPs, the cascade reaction converted glucose to gluconic acid. As a result, the surrounding pH decreased, creating a proton gradient. Subsequently, protons diffused to the lipid membrane-coated surface of HSM to facilitate ATP synthesis by ATP synthase. Chen et al. established a novel microcompartment structure using single-layer crosslinked zwitterionic vesicles (cZVs) with carboxyl-saturated cavities [39]. CeO_2_ nanozyme (SOD mimic) combined with Pt nanozyme (CAT mimic) could be synthesized in situ within the cavity. In vivo experiments demonstrated that this artificial organelle had a good therapeutic effect on ROS-induced ear inflammation.

**Figure 7:**
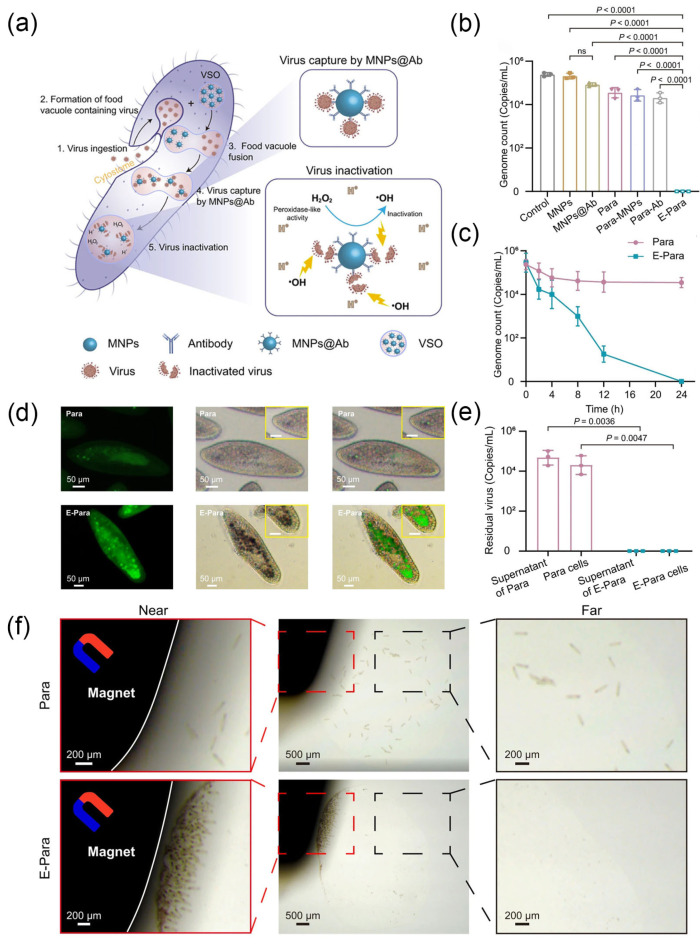
(**a**) The proposed mechanism of virus captures and inactivation by E-Para. (**b**) Virus capture capacity. (**c**) Time-dependent virus capture by Para and E-para. (**d**) Fluorescence images of Para and E-Para stained with HPF (a ·OH probe with green fluorescent). (**e**) Viral genome remaining inside E-Para or Para cells after treatment. (**f**) Recovery of Para and E-Para with a magnet. Reprinted with permission from Ref. [7]. Copyright (2023) Springer Nature.

Artificial membrane structures have consistently fallen short in terms of permeability and systemic coordination when compared to natural cell membranes [146]. Li et al. utilized vacuoles generated during the feeding process of *Paramecium caudatum* (Para) as microcompartment structures to create a semiartificial organelle capable of effectively clearing waterborne viruses (Figure 7a) [7]. Although some studies report that Para can ingest certain types of viruses, the virus capture and elimination capabilities are virus specific and less effective [147]. Therefore, it is not feasible to directly use Para as a strategy for virus removal. The authors designed Fe_3_O_4_ magnetic nanoparticles (MNPs) modified with virus-targeting antibodies (MNPs@Ab) and integrated them into the vacuoles of Para via a feed process to introduce a specific virus targeting and scavenging organelle (VSO). Subsequently, engineered Para (E-Para) with VSO, after ingesting viruses, fused vacuole-loaded viruses with VSO, allowing the viruses to be captured by MNPs@Ab (Figure 7b,c). Inside the VSO, the presence of a significant amount of hydrogen peroxide (H_2_O_2_) in the acidic environment stimulates the peroxidase-like activity of MNPs, generating ·OH, which effectively inactivate the virus (Figure 7d,e). After capturing the viruses, E-Para could be efficiently collected by external magnets for recovery and reuse, avoiding further contamination (Figure 7f). Finally, the authors replaced the antibodies on MNPs@Ab with sialic acid [148], enabling the simultaneous capture of three different viruses in water, demonstrating the universality of this strategy.

When artificial organelles are used in vivo, the responsiveness of microcompartment structures to the physiological environment poses a challenging strategy. This can be achieved by employing environmentally responsive materials [149]. Che et al. developed pH-sensitive polymer vesicles loaded with horseradish peroxidase (HRP) and urease [8]. The addition of acidic urea solution caused an instantaneous increase in the size and permeability of the polymer vesicles, allowing substrates to penetrate the polymer vesicles and initiate the catalytic process of HRP. As urea converted to ammonia, raising the pH, the polymer vesicles contracted, leading to the conclusion of the catalytic process. By providing acidic urea solution, the non-equilibrium nanoreactor could be restarted for multiple cycles. Outer membrane proteins (KcsA, OmpF, AqpZ, etc.) can serve as gates on the membrane structure, allowing substrates to enter the cavity and are typically inserted into liposomes to enhance permeability [150,151]. Furthermore, Einfalt et al. introduced molecular caps responsive to the redox environment into mutants of OmpF, enabling the diffusion of substrates into the membrane in oxidizing environments, making artificial organelles activatable by cellular glutathione levels [40]. The authors validated in vivo functionality in zebrafish embryos, demonstrating the feasibility of using artificial organelles as cell implants.

## 4. Three-Dimensional Multicellular Immune Niche

Multicellular communications play a pivotal role in coordinating a myriad of processes throughout tissue homeostasis and immune responses [152]. Innovations in biomaterials have allowed for the emergence of novel three-dimensional (3D) multicellular structures for immune modulation. Three-dimensional biomaterials with sufficient mechanical strength to withstand tissue pressure, adequate porosity for the ingress and egress of immune cells, and the ability to provide spatiotemporal control over biological signals to regulate immune cell function can recruit a diverse array of immune cells, including granulocytes, monocytes, DCs, macrophages, T cells, and B cells, forming a “multicellular immune niche” [14]. The in situ reprogramming of host cells and the loading of specific antigens enhance systemic anti-tumor immunity and allow for the establishment of an immunogenic microenvironment, providing opportunities for the continuous delivery of immune modulators or cells at specific locations [153]. Therefore, they are referred to as artificially synthesized “3D multicellular immune niche”.

Multifunctional scaffolds, such as hydrogels, frozen gels, mesoporous silica rods, provide a localized reaction site for storing immune cells and create a microenvironment to initiate immune responses. With the collaborative advantages between material chemistry and immunology, multifunctional material scaffolds hold tremendous potential for clinical translation [14,154]. In this section, we first discuss the methods for recruiting antigen-presenting cells (APCs) as the “cornerstone” for initiating immune responses, then introduce material scaffolds that ensure the rapid migration of immune cells within them, and finally highlight immune ecological niches of multicellular interactions, aiming to enhance immune effects.

The innate immune system detects, engulfs, and processes potential harmful antigens through APC, presenting them to naïve T cells to initiate adaptive immune responses [155]. Dendritic cells (DCs), as specialized “sentinels” of the immune system, play a crucial role both in initiating protective immunity against invading pathogens and inducing immune tolerance to harmless antigens, therefore bridging the innate and adaptive immune systems [156,157].

**Figure 8:**
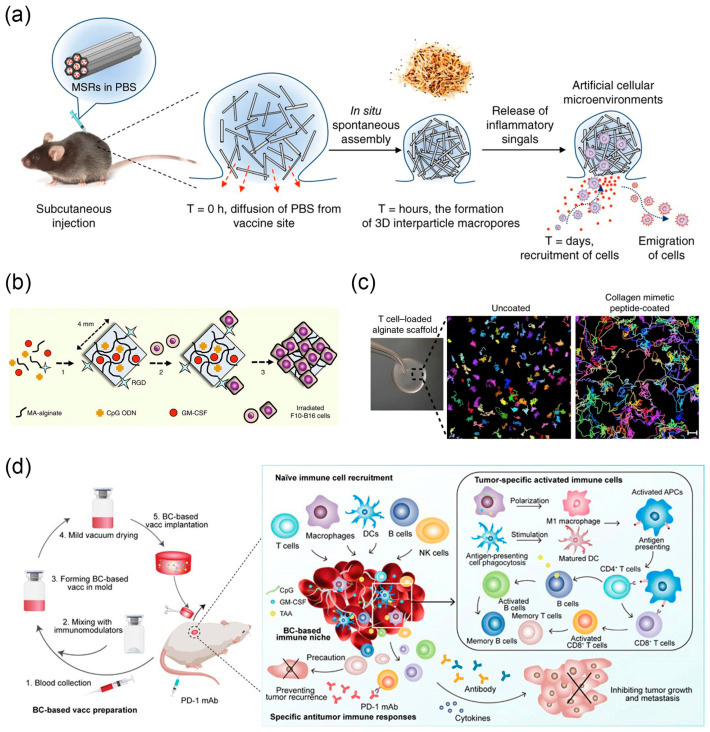
(**a**) A schematic representation of in vivo spontaneous assembly of MSRs and recruitment of host cells for maturation. Reprinted with permission from Ref. [41]. Copyright (2015) Springer Nature. (**b**) Fabrication of irradiated tumor cell-loaded cryogel sponge vaccines. Reprinted with permission from Ref. [160]. Copyright (2015) Springer Nature. (**c**) Time-lapse video projections of lymphocyte migration. Reprinted with permission from Ref. [45]. Copyright (2015) Springer Nature. (**d**) Schematic of implantable blood clot vaccine. Reprinted with permission from Ref. [46]. Copyright (2020) American Association for the Advancement of Science.

In the early stages, researchers harnessed the natural migratory ability of APCs to design scaffold materials. By releasing signals such as cytokines to create chemotactic gradients, they recruited DCs and provided antigens and other danger signals to activate and enhance the homing of DCs to lymph nodes [158]. Fenton et al. designed an injectable hydrogel that comprises hydroxypropyl methylcellulose (HPMC) polymer-poly(ethylene glycol)-block-polylactide (PEG-b-PLA) nanoparticles which is shear thinning and self-healing to ensure the injectivity of the gel. Through the sustained release of the cytokine CCL21, this hydrogel recruited DC cells [159]. In a related approach, subcutaneous injection of high aspect ratio mesoporous silica rods (MSRs) can self-assemble into large pore structures in vivo which are loaded with the antigen OVA, a potent stimulator of dendritic-cell recruitment and proliferation granulocyte-macrophage colony-stimulating factor (GM-CSF), and a potent agonist of eliciting strong CD8 killer T cell-mediated immune responses unmethylated cytosine-phosphate-guanine oligonucleotide (CpG-ODN) sequence (Figure 8a). Compared to polymer scaffolds, MSRs significantly increased the recruitment of DC cells, generating effective humoral and cellular immune responses [41,42].

To avoid surgical implantation, Mooney et al. developed a highly compressible freeze gel that rapidly regains its original shape upon subcutaneous injection. Freeze gels are typically made from Gelatin methacryloyl (GelMA) [161], alginate [162], polyethylene glycol (PEG) [43], and other modified polymers. They utilize ice crystals formed during polymerization at sub-zero temperatures to create interconnected large pores. Covalently linked alginate freeze gels with RGD peptides enhanced their adhesion to encapsulated tumor antigens [160]. Controlled release of DC recruitment factor GM-CSF and immune activation factor CpG ODN increases the number of recruited DC cells and effectively activates these recruited cells (Figure 8b). Subsequently, within the microenvironment provided by the freeze gel, DCs interact with the loaded tumor antigens, and trigger an effective, durable, and specific anti-tumor T cell response. Their group’s further research combined covalent and ionic crosslinking to prepare methacrylate (MA)-alginate freeze gels [163]. These gels exhibited higher mechanical strength and could be injected with smaller needle diameters. Moreover, the distance from the injection site of the cryogel to the draining lymph nodes does not significantly impact the homing kinetics of DC cells and the subsequent speed and intensity of the downstream antigen-specific immune response [44].

The immunotherapy that recruits APCs using biomaterials can also combine with other therapies to enhance immune responses. Fan et al. proposed a thermosensitive hydrogel loaded with in situ formed manganese dioxide nanoparticles (MnO_2_ NPs) [164]. Through photothermal therapy (PTT), it induces immunogenic cell death in autologous tumor cells, releasing a large amount of tumor-derived antigen proteins. The introduction of strongly adhesive groups like catechol provides the hydrogel with the ability to adhere to antigens, acting as an “antigen reservoir”, so that it can continuously release antigens, recruit more dendritic cells, and stimulate a robust and persistent anti-tumor immune response mediated by CD8 T cells.

Both antigen-specific T cells in the initial immune response and dendritic cells (DCs) in lymphoid organs exist at low frequencies. The formation of a long-term stable immune synapse between T cells and DC occurs only when T cells recognize DC presenting homologous antigens, leading to T cell activation; otherwise, T cells do not linger for an extended period. Thus, T cells must move rapidly within the body to scan antigen-presenting cells (APCs), search for and monitor antigens, and respond to them, aiming to eliminate pathogens and tumor cells at the earliest stage [165]. Therefore, scaffold materials that support the rapid migration of APCs and T cells have emerged.

Cell migration is regulated by the cell adhesion capability of the surrounding environment and chemotactic factors inducing movement. Hydrogels, due to their controllable mechanical properties and structural characteristics, not only serve as physical scaffolds for loading cells but also can control the release of stimuli signals to regulate cell behavior, making them a common platform for studying the interaction between cells and their microenvironment [166]. Stachowiak et al. used internally ordered, interconnected, mesh-like porous PEG hydrogel as a substrate, infiltrated with collagen and adhesive protein ICAM-1, to support the migration of T cells and DCs within the scaffold [167]. Further integration of the chemotactic factor CCL21 onto the gel wall induced gradient movement of T cells inside the structure, creating an engineered microenvironment that supports local T cell responses. Stephan et al. integrated artificially synthesized collagen-mimetic peptide GFOGER onto large-pore polyacrylate scaffolds using carbodiimide chemistry to facilitate lymphocyte rapid migration along collagen fibers [45]. Using a time-lapse microscope confirmed that T cells migrated on the scaffold at a speed similar to that in lymphoid organs (119 μm ± 37 μm within 30 min), while lymphocytes in unmodified scaffolds moved only within their gap spaces (7 μm ± 4.8 μm within 30 min) (Figure 8c). Simultaneously, silicon dioxide microspheres were encapsulated into the scaffold gap spaces. Their surfaces were coupled with signals required for T cell activation and proliferation, such as anti-CD3, anti-CD28, and anti-CD137 antibodies. As a result, a biopolymer implant capable of supporting rapid migration and robust expansion of T cells was constructed. T cell proliferation rate increased by 22 times, and the number of T cells migrating towards the surrounding collagen gel significantly increased.

Engineered scaffolds with defined mechanical, physical, and chemical properties can reprogram the local immune microenvironment, providing sites for immune cell interactions and amplification [168]. By residing for extended periods in targeted areas such as tumors, these scaffolds can more precisely manipulate directed immune responses. They have the potential to mimic or reconstruct natural extracellular matrix-like structures and become an ideal customizable platform for individualized therapy after loading specific antigens [169]. In addition, compared to systemic immune modulation, local immune modulation can achieve more effective treatment at lower doses, preventing systemic toxicity and disruption of homeostasis.

In 3D biomaterials, hydrogels exhibit tunable swelling properties and similarity to the extracellular matrix (ECM), offering an ecological niche that permits cell ingress and egress [170]. Particularly, injectable hydrogels formed in situ present a simple operational approach, effectively avoiding local trauma caused by surgery, holding great promise in the field of vaccine development [15]. Inspired by the blood clotting process, Wang et al. developed an autologous blood clot scaffold (Figure 8d) [46]. The fibrin network formed during blood clotting captures red blood cells, creating a blood clot scaffold. This scaffold can recruit various immune cells, forming an “immune niche” that induces innate immune responses. Further loading tumor antigens and adjuvants CpG ODN, GM-CSF into the blood clot scaffold stimulated the recruited immune cells into tumor antigen-specific immune cells, effectively inhibiting the growth of B16F10 and 4T1 tumor, establishing an effective personalized cancer vaccine. Programmed death ligand PD-L1 exists on the surface of local tumor cells or the surface of their circulating exosomes. It binds to the programmed death receptor PD-1 on the surface of lymphocytes, leading to lymphocyte depletion and achieving immune suppression. Li et al. created a PD-L1 checkpoint-regulated immune niche using injectable hydrogels [171]. Oxidized sodium alginate (OSA)-armored tumor membrane vesicles (O-TMVs) injected into the tumor region, in situ chelating Ca^2+^ converted into a hydrogel serving as an antigen reservoir, recruited various immune cells to form an immune niche. Cyclin-dependent kinase 5 (Cdk5) inhibitor roscovitine and Ca^2+^ channel inhibitor dimethyl amiloride (DMA) were simultaneously encapsulated in the hydrogel, obtaining O-TMV@DR hydrogel. Roscovitine can inhibit the expression of PD-L1 on tumor cells and their secreted circulating exosomes. Ca^2+^ depletion induced by OSA chelation, in synergy with DMA, inhibits exosomes secretion by reducing intracellular Ca^2+^ in tumor cells [172]. Therefore, O-TMV@DR hydrogel can effectively inhibit tumor growth and metastasis, providing a new approach for immune checkpoint therapy. Phuengkham et al. achieved joint spatiotemporal modulation of tumor-derived immune suppression and vaccine-induced immune stimulation through an artificially synthesized immune niche [173]. They constructed a 3D scaffold crosslinked with collagen and hyaluronic acid, capable of continuously releasing gemcitabine to alleviate tumor immune suppression and delivering a cancer vaccine containing whole tumor cell lysates and polyinosinic:polycytidylic acid (poly(I:C)) nanoadjuvants to enhance the immune response. The result was the generation of systemic anti-tumor immunity, achieving a 100% survival rate.

**Figure 9:**
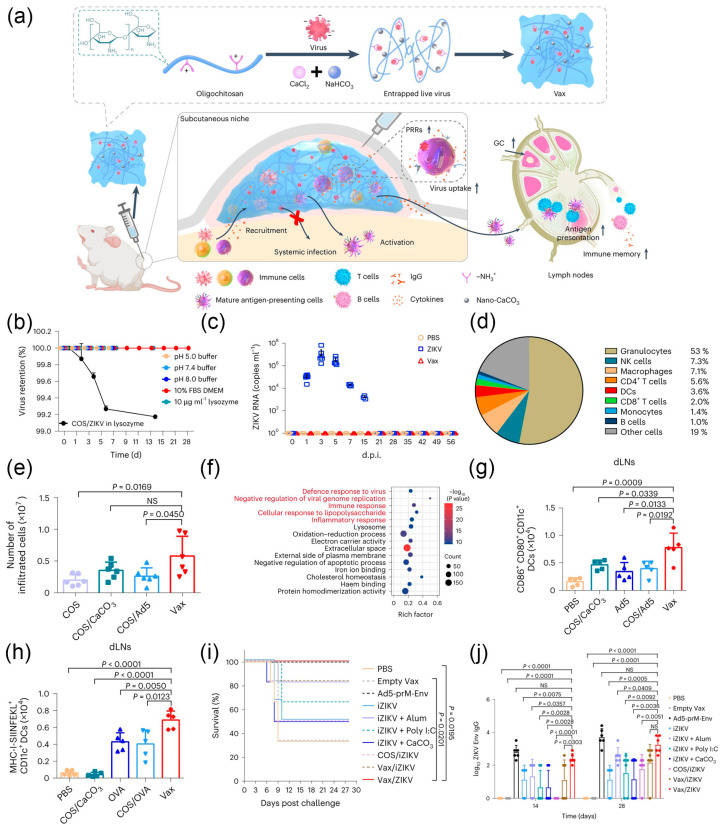
(**a**) Schematic of the virus-entrapping hydrogel. (**b**) Vax/ZIKV was immersed in various solutions to see the release of virus. (**c**) Viraemia of surviving IFNAR1^−/−^ mice after infection. (**d**) The frequency of cells recruited in vivo. (**e**) The number of infiltrated cells from the retrieved nodules. (**f**) Kyoto Encyclopedia of Genes and Genomes pathway analysis of DEGs of RAW 264.7 cells. (**g**) Number of CD80^+^ CD86^+^ CD11c^+^ cells at 2 d post immunization. (**h**) Number of DCs presenting SIINFEKL on the MHC-I molecule at 7 d post immunization. (**i**) Survival curves of immunized IFNAR1^−/−^ mice after lethal challenge with ZIKV. (**j**) The production of anti-ZIKV-Env IgG immunized for 2 and 4 weeks. Reprinted with permission from Ref. [16]. Copyright (2023) Springer Nature. NS: not significant.

In addition to anti-tumor immunity, 3D multicellular immune niches have been employed in the study of antiviral immunity. Encapsulating viruses in material scaffolds enables the local aggregation of immune cells while preventing leakage, thus constructing an immune factory for the temporal and spatial regulation of interactions between viruses and immune cells. In light of this, Hao et al. proposed a strategy to convert live viruses directly into vaccines using hydrogels. They used wild-type Zika virus (ZIKV) as a model and prepared a live virus-embedded hydrogel named Vax (Figure 9a) [16]. Positively charged chitosan (COS) was employed to immobilize negatively charged ZIKV virus particles. The gelation characteristics of Vax at 37 °C effectively prevented virus leakage and escape, validated through in vitro release and infection experiments in IFNAR1^−/−^ mice (Figure 9b,c) [174]. Furthermore, the inclusion of inorganic calcium carbonate nanoparticles (nano-CaCO_3_) in Vax prevented the degradation of the gel structure in acidic environments and served as a stable Ca^2+^ source, promoting the rapid recruitment of various immune cells at the injection site to form an immune niche (Figure 9d,e) [175]. In vivo injection of Vax upregulated pattern recognition receptors (PRRs) of innate antiviral immune responses (Figure 9f), and facilitated immune cell infiltration into the hydrogel for virus engulfment, antigen processing, and cross presentation (Figure 9g,h). Subsequently, immune cells homed to lymph nodes, inducing germinal center (GC) responses, further generating an efficient systemic immune response. After subcutaneous immunization in mice for 28 days followed by viral challenge, the survival rate increased to 100%, demonstrating effective protective effects (Figure 9i,j). This strategy not only provides a new approach for the design of viral vaccines but also exhibits a degree of universality, holding promise for the development of emergency vaccines.

Compared to traditional hydrogels, 3D printing enables large-scale, cost-effective, and stable manufacturing. Zhang et al. developed a porous and ordered scaffold based on gelatin and sodium alginate [47]. Various recruited immune cells were trained with tumor antigens and adjuvants for specific targeting of tumor cells. Compared to traditional hydrogels, the 3D scaffold with well-organized porous structure imparts functionalities similar to real lymphoid organs, forming an “artificial tertiary lymphoid structure”. Postoperative inhibition of tumor recurrence models highlighted the advantages of 3D printing. Dang et al. proposed an implantable gelatin-sodium alginate hydrogel scaffold incorporating CuO nanoparticles [48]. The size and shape-controllable 3D-printed scaffold could fill the defect site after surgical resection, providing mechanical support to the tissue. During in vivo degradation, the scaffold continuously released CuO nanoparticles, suitable for photothermal therapy and capable of reacting to generate Cu^2+^ under acidic conditions. Cu^2+^ induces intracellular reactive oxygen species (ROS) through a Fenton-like reaction, leading to ferroptosis by depleting glutathione (GSH) and inactivating GPX4. This multifunctional system presents a novel approach to develop an implantable platform for postoperative tumor recurrence inhibition.

## 5. Conclusions and Outlook

Carefully designing the physicochemical properties of biomaterials and exploring the synergistic effects among various biomaterials can yield unexpected and modularly diverse functions of living organisms. Particularly, using environmentally responsive biomaterials, such as the artificial organelles with pH-responsive switches [8] and the artificial coat that enable cells to respond to glucose gradients for directed movement [176], can achieve controlled interactions between living organisms and their external environment. It can be reasonably expected that the combination of complex and diverse biomaterials will help us construct more sophisticated components for living organismal modifications. While exploring new possibilities, researchers should also be mindful of the complexity of living organisms. Currently, there is a lack of biochemical understanding of the interactions between biomaterials and living organisms and their biological consequences. The relevant study of mechanisms will not only contribute to better enhancing and modifying living organisms with biomaterials but also promote the development of fundamental disciplines in biology. Together, biomaterial-promoted functional enhancement of living organisms is a fascinating and rapidly advancing field. This strategy draws inspiration from the diversity of natural organisms and utilizes the physicochemical properties of biomaterials for artificial enhancements of living organisms, facilitating advancements in cell protection, cellular delivery, cancer treatment, and vaccine improvements. These studies will contribute novel insights to the field of chemicobiology.

## Figures and Tables

**Table 1:** An overview of three strategies to enhance organismal functions through biomaterial intervention.

Strategies	Organisms	Functional Enhancement	Main Biomaterials	References
Biointerface engineering	JEV/EV71	Thermal stability	CaP	[18,19]
	EV71	Thermal stability	Aluminum oxide	[20]
	DENV	ADE effects elimination	CaP	[21]
	Ad5	Thermal stability	ZIF-8	[22]
	MSCs	Tumor targeting	Gelatin	[23]
	Yeast cells	Protection	Polymers, CaP	[24]
	Hela	Protection	Silica	[25]
	Jurkat T	Protection	TiO_2_	[26]
	Green algae	Hydrogen production	Silica	[27]
	Yeast cells	Catalysis	ZIF-8	[28]
	Probiotics	Protection	Fe^3+^-TA	[29]
	Jurkat T	Functional group	Polymers	[30]
	Cancer cells	CCTC	CaP	[31]
	Macrophages	Drug delivery	Nanosponges	[32]
	RBCs	Blood transfusion	Polymers	[33]
Artificial organelles	Cancer cells	Prodrug therapy	HSN	[34]
	Macrophages	Catalysis	Polymers	[35,36]
	Kidney cells	Antioxidants	RuO_2_	[37]
	Normal cells	Chemotherapeutic drug capture	Au-ODN	[38]
	Normal cells	Inflammation clearance	cZV, CeO_2_	[39]
	*Paramecium caudatum*	Waterborne virus clearance	Fe_3_O_4_	[7]
	Zebrafish embryos	Stimuli responsive	Liposome	[40]
3D multicellular immune niches	DCs, T cells	Immune responses	MSRs	[41,42]
	DCs	Recruitment, activation	Freeze gel	[43,44]
	T cells	Migration	PAA, collagen fibers	[45]
	Immune cells	Recruitment, activation	Blood clot	[46]
	Immune cells	Antiviral immunity	Chitosan	[16]
	Immune cells	Immune responses	3D-printed scaffold	[47,48]

## Data Availability

Data are contained within the article.
